# Are equity, diversity and inclusion considered in early-phase studies evaluating innovative and developing surgical procedures? Protocol for a scoping review

**DOI:** 10.1136/bmjopen-2025-112489

**Published:** 2026-02-09

**Authors:** Manal Etemadi, Rhiannon Macefield, Kerry Avery, Daisy Elliott, Shoba Dawson, Natalie S Blencowe, Maeve Coyle, Hollie Sarah Richards, Benjamin Graham, Eddie Jones, Amber Torkington, Katie Chatfield, Hussein Malik, Mackenzie Garlick, Jane Blazeby

**Affiliations:** 1 Bristol Centre for Surgical Research, Bristol Medical School, Department of Population Health Sciences, University of Bristol, Bristol, UK; 2 NIHR Bristol Biomedical Research Centre, University Hospitals Bristol and Weston NHS Foundation Trust and University of Bristol, Bristol, UK; 3 School of Medicine and Population Health, The University of Sheffield, Sheffield, UK; 4 Warwick Medical School, University of Warwick, Coventry, UK; 5 Bristol Medical School, University of Bristol, Bristol, UK

**Keywords:** Protocols & guidelines, SURGERY, Health Equity, Review

## Abstract

**Abstract:**

**Introduction:**

Increased risks and concerns regarding patient safety in early-phase studies exist because knowledge about the new intervention is still accumulating. This means that narrow eligibility criteria are needed. However, if early-phase studies are narrow in their inclusion, for example, by not including diverse populations, there is a potential risk that new therapies have insufficient relevant efficacy and safety data. Existing research has explored equity, diversity and inclusion (EDI) factors in early-phase pharmaceutical studies, but it has not been possible to find studies that have systematically examined whether EDI factors have been considered in surgical studies reporting innovative procedures. We aim to examine how EDI factors are considered in early-phase surgical studies and surgical innovation reports to explore how this may impact on later-phase evaluation and inclusive intervention implementation.

**Methods and analysis:**

A scoping review following the JBI (Joanna Briggs Institute) and Arksey and O’Malley’s five-step process is being conducted. We will search Scopus, PubMed and Web of Science for surgical early-phase studies. A two-step screening process for eligibility is being used. Independent double screening will take place for 20% of the papers. Eligible articles will report early evaluation of an innovative surgical/invasive procedure. Excluded will be comparative and later-phase studies and early evaluations of pharmaceutical products even in a surgical setting. Data on article details, patient eligibility and whether protected characteristics are reported and considered will be extracted. Information about EDI considerations reported in the introduction or discussion of the papers will also be extracted. Findings will be discussed with a patient advisory group. A content synthesis approach will be undertaken and descriptive summaries presented.

**Ethics and dissemination:**

This study does not require ethical approval being a secondary analysis. The findings will be disseminated through academic journal publications and oral presentations.

**STRENGTHS AND LIMITATIONS OF THIS STUDY**

- The review follows established scoping review guidance, including the Joanna Briggs Institute and Arksey and O’Malley frameworks.
- Data extraction and reporting are informed by Preferred Reporting Items for Systematic Reviews and Meta-Analyses 2020 items and expert input from a multidisciplinary team at the Centre for Surgical Research and a librarian.
- Peer-reviewed literature from three electronic databases will be included due to the high volume of early-phase studies.
- Grey literature will not be included, which will allow us to map how equity, diversity and inclusion factors are considered in original research.
- The review is limited to English-language publications, potentially introducing language bias.

## Introduction

 Surgical innovation has a critical role in improving patient care and in optimising use of health resources. Innovation ranges in magnitude from being something ‘completely new’, through to ‘a major modification’, or to being ‘not at all new’. All carry different magnitudes of risk.^1^ The variation in part reflects the complexity of surgical interventions that encompass multiple technical components, linked co-interventions before and after surgery, and operator and team skill. One or more, or all of these components may be new or modified with associated added and unknown risks. The innovation pathway therefore requires comprehensive evaluation and risk assessment. Within research settings, innovation occurs within early-phase studies. Innovation also occurs in clinical practice outside of research, typically reported in single case studies and case series. Innovative surgical interventions may have approval from local hospitals, be registered as audits or quality improvement projects or may not be registered and have governance oversight.^2^ These studies or projects all allow exploration of technical modifications, patient eligibility, safety and efficacy,^3 4^ although the latter are less well documented than their research counterparts. When promising innovations are introduced into clinical practice, there is typical progression from small single centre studies to multicentre studies, and some progress directly to implementation or full later evaluation within a definitive (phase 3) randomised trial.

Choice of participants, number of participants to be studied, safety assessments and stopping rules should be thoroughly considered and decisions justified and explained in study protocols.^5^ Reporting is also important to allow transparency. However, the overall reporting quality of early-phase studies in this area is often suboptimal.^6^ One aspect that is frequently poorly reported is the rationale and details for patient eligibility and exclusion.^7^

The increased risks and concerns regarding patient safety in early-phase studies stem from there being a lack of prior evidence for the new procedure. It is therefore critical that eligibility criteria are carefully considered, and usually narrow criteria are used.^8 9^ The drawback to this safe approach is that if early-phase studies do not include diverse populations the accumulating data are likely to lack generalisability.^10^ This applies to all patient characteristics, for example, sex, ethnicity and disability status. Therefore, the balance between safety and inclusion is important and such issues require further consideration in the incremental development and evaluation of new procedures.

Equity, diversity and inclusion (EDI) concern the inclusion of underserved groups in research. Equity relates to ensuring that there is equal access for people to the same opportunities, regardless of personal characteristics and experience. Diversity in research is the practice of optimising inclusion of participants from different backgrounds. Inclusion relates to creating an environment where everyone feels valued.^11^ EDI components/dimensions such as ethnicity and gender are factors that require consideration in clinical research to ensure that the evidence created is relevant for the intended populations.

Research in EDI considerations in early phase studies is limited and what is known primarily comes from clinical areas other than surgery, predominantly early phase studies within pharmaceutical science. People from ethnic minority groups and women are often overlooked and under-represented.^12^,^15^ It is still not standard for research findings to separately report by sex or gender.^16^ Other factors such as age and language skills may limit participation in early-phase studies.^17 18^ These factors are important as sometimes subgroups of patients have different responses to treatments compared with others. For example, people in some ethnic groups metabolise certain drugs quite differently compared with others.^19^ Although some publications in this field are available about ethnicity much less is known about other protected characteristics.^20^

People living with disability and comorbidities (more common in socio-economically deprived groups) are less likely to be referred for inclusion in early phase cancer trials.^21^ Nonetheless, there is a debate that suggests minorities might be over-represented in some studies such as those including healthy volunteers, because participants use research to supplement income and make this role an occupation, ‘a professional guinea pig’.^22^,^25^ There are others who have noted, however, that people in minority groups fear research participation because of the risk of being treated like a ‘guinea pig’.^26^

EDI issues may be particularly relevant for surgical research. For example, studies have shown differences in patients’ perceptions and experiences of scarring and wound recovery across ethnic groups, with implications for psychosocial well-being and equitable postoperative care,^27^ and recovery from surgery in people with disabilities may be more complex.^28^ Evidence shows protected characteristics are under-reported in phase 3 trials in colorectal cancer resection.^29^ It has not been possible to find studies that have systematically examined whether EDI factors were considered in surgical studies reporting innovative procedures.

### Study aim

The aim of this scoping review is to examine if and how EDI factors are considered in early-phase surgical studies and surgical innovation reports and to explore how this may impact on later-phase evaluation and inclusive intervention implementation.

## Methods

### Scoping review

We aligned our methods with the Joanna Briggs Institute approach for scoping reviews.^30^ This involves a five-step process (identifying the research question, identifying relevant studies, selecting studies, charting the data and collating, summarising and reporting the results).^31^ The Preferred Reporting Items for Systematic Reviews and Meta-Analyses (PRISMA) extension guidelines for reporting scoping reviews will be followed.^32^

### Identifying the initial research question

Our research question aims to understand whether EDI factors were considered in surgical early phase studies. We are therefore interested in whether any of the surgical early phase studies reported EDI factors and understanding what EDI factors are reported. These are the key questions:

1. In published surgical early phase studies and innovation reports, have authors reported EDI factors?
2. What EDI factors did the studies report in their recruitment?
3. Did authors consider EDI implications for the next stage of research?

### Identifying relevant studies

We aim to identify early phase studies of innovative surgical and invasive procedures. There are no existing standard search terms for these types of studies. We have developed a search strategy based on existing reviews of early-phase studies.^33 34^

We will search the Scopus, PubMed database and Web of Science platform for published early-phase studies and innovation reports using guidance from a research librarian. Table 1 shows terms used to build the search strategy. The online supplemental appendix shows the full strategy. Where appropriate, title/abstract/key words will be applied to the databases. The keywords within each key search term will be separated by the ‘OR’ Boolean operator. The innovation and surgical procedure key terms will be separated by the ‘AND’ Boolean operator. Full electronic search strategies for each database are shown in the online supplemental appendix 1.

**Table 1:** Keywords used to identify literature

Key terms	
Innovation	Early-phase, phase 1, phase 2, innovative, transformative, new, novel, initial, experimental, emerging, IDEAL, feasibility, first case, initial case, first in human, first in human, preliminary, proof of principle, case reports, pilot studies.
Surgical procedure	Invasive, incision, percutaneous, surgery, operat*, interventional, surgical procedures.

* indicates a truncation symbol for the search terms - to retrive variations of words starting with the word stem 'operat'

### Study selection

Table 2 presents the eligibility criteria. This includes:

1. Surgical/invasive procedures.
2. Early-phase studies and innovation reports expressing innovation with words like new, innovation, novel, first time, etc or specific terms such as phase 1 and 2, IDEAL 1, 2a/2b.
3. Original research indicating recruiting patients for the specific intervention.

**Table 2:** Eligibility criteria for included papers

Inclusion criteria	Early-phase study Case series and prospective patient studies of innovative procedures reporting safety and efficacy data (non-comparative). Single patient case studies of innovative procedures. Patient studies self-reported as phase 1, 2a, 2b. Patient studies self-reported as Ideal stage 1, 2a, 2b: IDEAL 2b will still be non-comparative, but if there are IDEAL 2b and comparative—then they will not be included. All the above are considered prospective by design.	An innovative procedure Text that describes a procedure as new, modified or innovative eg, novel, first in human. Text that describes the procedure to have uncertain safety or efficacy. Text that describes the procedure is being done for the first time in a different place eg, first in a specific country. Text that describes the first time the procedure is being performed in a different patient group eg, first time in children (usually do not in adults). Text describing the procedure being done for the first time by a different type of clinician eg, first-time radiologists do an invasive procedure normally done by surgeons.	A surgical or invasive procedure procedure that involves deliberate access to the body through an incision, percutaneous puncture or natural orifice. If a medicinal product is administered as part of an invasive procedure, it qualifies as invasive when operator skill is needed to deliver it internally or to a specific anatomical area.^37^ This includes all these: Traditional ‘surgical’ interventions. All minimal access eg, laparoscopic, robotic. All endoscopy with therapeutic interventions (not just diagnostic). All PCI with therapeutic interventions (not just diagnostic).
Exclusion criteria	All later-phase evaluations (eg, phase 3, all RCTs, registry studies, epidemiological data analyses, comparative non-randomised studies), and commentaries, letters, editorials and audits, systematic review and meta-analysis, economic evaluations, retrospective studies, technical notes and surveys. Non-therapeutic invasive and imaging procedures. Animal studies and pharmaceutical studies. Publications in the non-English language. Studies that explicitly report a retrospective design. Grey literature (including conference abstracts and dissertations).		

PCI, Percutaneous coronary intervention; RCT, Randomised Controlled Trial.

Due to expected high number of studies, we will limit the date of publication by a period within year 2025 (January to April). This is to ensure the manageability of the screening process, and to allow the review to focus on the most recent innovations, including emerging trends and the most up-to-date reporting practices in the field.

In single-patient case studies, EDI dimensions will be recorded based on how patient characteristics are described and whether authors reflect on implications for broader populations or for equitable access to the innovation.

Final search results will be imported into a software tool for assisting with screening for reviews (Rayyan)^35^ where they will be deduplicated and screened. A team will undertake the screening with iterative training and meetings in which eligibility criteria will be explained and discussed to reach a shared understanding. First, this initial subset, comprising 20% of the total records will be independently double screened by two reviewers to ensure the shared understanding and alignment on inclusion and exclusion criteria. Each pair of reviewers will complete the screenings independently in Rayyan. A third reviewer will review these and resolve records where there is disagreement. The remaining 80% of the references will be allocated across all reviewers for single independent screening with any uncertain or borderline records discussed with a second reviewer.

Second, full text articles will be retrieved for those categorised as potentially eligible and reviewed by two researchers independently. Meetings will be held to discuss and clarify where disagreements exist, with a third reviewer making final decisions as needed. This whole process will be presented as a PRISMA flow diagram.

### Charting the data

An Excel data extraction sheet will be created to capture details about: study characteristics (basic information about the study, country of origin, study aim, surgical specialty, stage of innovation and study design), population characteristics and EDI (on patient eligibility and distribution of protected characteristics including age, sex, gender, sexual orientation, race, disability, socioeconomic factors, etc)^36^ and further EDI considerations which imply any justification in introduction and/or discussion on the rationale behind the recruitment and any explanation for future and later-phase studies. Table 3 shows the data extraction items.

**Table 3:** Plans for data extraction from included full text articles

Topic	Question
Study characteristics	
Title	Title of the article
Journal	Name of the journal the study is published in
Authors	Name and contact information of all authors
Country of origin	What is the country of origin based on the first author’s affiliation with the paper?
Objective	Aim or research question
Country of origin	What is the country of origin based on first author affiliation of the paper?
Surgical specialty	General surgery, plastic, vascular, neurosurgery, ophthalmology, ENT, cardiothoracic, urology, orthopaedics, gynaecological, colorectal, paediatric, multiple and other
Surgical procedure	What were the specific surgical procedure(s)?
The innovation	It there text that supports this procedure/intervention being innovative?
Stage of innovation	What is the stage of innovation?
Study design	What is the author-reported study design?
Ethics approval	Is there any mention of ethics approval, eg, HRA, Institutional Review Board, …?
Population characteristics and EDI	
Recruited sample size	What is the total recruited sample size?
Number of recruiting centres	What is the number of recruiting centres?
Inclusion and exclusion criteria	Did the paper report inclusion criteria? Did the paper report exclusion criteria?
Protected characteristics (age, sex, gender, sexual identity, race/ethnicity, socioeconomics factors, education level, disability, location)	What is the age range of participants as specified in the result? Did the paper report sex distribution as specified in the result? (male, female, intersex, NR*) Did the paper report gender categories? (man, woman, both, other, NR) Did the paper report sexual identity†? (heterosexual, bisexual, gay, lesbian, transgender, NR) Was race/ethnicity reported? Are any socioeconomic factors reported? (income level, employment, insurance, other, multiple, NR) Is education level reported? Is disability status reported? Are geographical regions of patients reported?
Further EDI considerations in the paper	
Implication in introduction/discussion	Did the study mention any reference to EDI in the introduction/discussion/other sections?
Safety by EDI factors	Did the paper report safety by EDI factors (eg, race)?
EDI-related anatomical variations	Are there any EDI-related anatomical variations between patients that have had the procedure reported?
Barriers to participation among minorities	Are barriers to participation among minorities mentioned?
Strategies for improving participant diversity	Are strategies for improving participant diversity mentioned?
EDI implications of findings	Did the study discuss the implications of findings for EDI?
Recommendations made for improving EDI	Are recommendations/plans made for improving EDI in future trials?
EDI in relation to stakeholder engagement in the innovation/study development	Did the paper mention EDI in relation to stakeholder engagement in the innovation development or the study development?

* NR: Not reported.

† Studies involving innovative gender reassignment surgical procedures will be eligible for inclusion if they meet our inclusion criteria.

EDI, equity, diversity and inclusion; ENT, Ear, nose and throat; HRA, Health Research Authority.

An iterative approach to updating the data extraction form will be taken. Initially, the extraction sheet will be piloted by the research team using five papers and a meeting to consider item interpretation. Data will then be extracted from the remaining articles, by one author with a second checking the entries. The final number of included articles will dictate how this is shared between reviewers.

Race/ethnicity, sex, gender, sexual orientation, age, language, religion, socioeconomic status (income level, insurance status, employment, etc), rural/urban population, comorbidities, disability, immigration status, as per the PRO EDI participant characteristics table on Trial Forge guidance will be explored.^36^

In this review, EDI will be operationalised through the identification and reporting of EDI dimensions or factors described within early-phase surgical studies. We treat these as socially constructed and context-dependent categories, recognising that they reflect social positioning and access to opportunities rather than inherent biological traits.

During data extraction, we will record whether and how these EDI dimensions are (1) reported (eg, demographic breakdowns, inclusion/exclusion criteria), (2) considered in study design (eg, recruitment strategies or sampling) and (3) analysed or discussed in relation to study outcomes. Where authors use alternative terminology or frameworks, these will be mapped to corresponding EDI dimensions to ensure consistency across studies.

### Quality assessment

Scoping reviews do not assess the quality of the published studies; rather, they aim to map the available evidence; therefore, critical appraisal of included sources is generally not required.

### Patient and public involvement

The results will be shared and discussed with our patient advisory group based in the National Institute for Health and Care Research (NIHR) Bristol Biomedical Research Centre.

### Collating, summarising and reporting the results

Summaries of the study characteristics will be presented in tables. Data will be described as frequencies (where possible) to provide a numerical overview of the quantity, nature and distribution of the included studies. This will be supplemented with a narrative summary using data categorisation as appropriate. The narrative synthesis will involve a description of the extracted baseline characteristics of the included studies including summaries of the included studies and the EDI considerations/factors.

The narrative synthesis of EDI reporting will summarise and describe the volume of studies that consider EDI issues at any level. For studies with some EDI consideration a content synthesis approach will be used to identify commonalities and differences between the studies, if possible. Reported EDI factors will be grouped in different ways to explore the patterns, and we will summarise and describe how studies with EDI considerations use these to describe how future evaluation considers EDI factors.

### Strengths and limitations

To our knowledge, this will be the first scoping review to explore whether surgical early-phase studies consider EDI factors in their participant recruitment and if so, which factors. It is strengthened by use of a systematic approach to identify and collect data informed by PRISMA 2020 guidance as well as guidance on conducting scoping reviews. This will be supplemented with input from the multidisciplinary team at the Bristol Centre for Surgical Research, evidence synthesis experts and a research librarian.

Due to the high volume of early phase studies, the search strategy only includes three electronic databases to allow us to pragmatically map how EDI factors are reported in original research. This review will be limited to publications in English; this may introduce bias against studies that may be published in other languages.

## Ethics and dissemination

No ethical approval is required as no primary data will be collected. The study will adhere to the protocol. The results will be reported in a paper and submitted for peer review and publication. It is also intended to present the findings at clinical and methodological conferences.

## Discussion

This report describes our planned scoping review of published surgical early-phase studies to explore their consideration of EDI in participants’ recruitment.

To the best of our knowledge no similar reviews exist. The protocol has been developed according to standard frameworks. Both numerical summary and narrative synthesis will be employed to relevant information from included studies. The first steps of the research have started and over 5000 abstracts have been identified for screening.

Methodological limitations of this review include the restriction to English-language publications which may introduce language bias and limit the global representativeness of findings. The exclusion of grey literature and conference abstracts may result in omission of emerging or unpublished evidence. In addition, the heterogeneity and variable reporting quality of early-phase studies may constrain the comparability of EDI-related data across studies.

Despite these limitations, this review will provide an important methodological foundation for understanding how EDI is currently addressed in surgical innovation and inform future efforts to improve inclusivity in early-phase research.

## Supplementary material

10.1136/bmjopen-2025-112489 online supplemental file 1
