# Supplementary material for: Are equity, diversity and inclusion considered in early-phase studies evaluating innovative and developing surgical procedures? Protocol for a scoping review
Source: BMJ Open. 2026 Feb 9;16(2):e112489. doi: 10.1136/bmjopen-2025-112489 (PMC12887517; doi:10.1136/bmjopen-2025-112489)
Supplement: online supplemental file 1 [file bmjopen-16-2-s001.docx]

Appendix 1: Search strategy

| **Data base** | **Search strategy** |
| --- | --- |
| PubMed | (("early phase"[Title/Abstract] OR "phase I"[Title/Abstract] OR "phase II"[Title/Abstract] OR "innovative"[Title/Abstract] OR "transformative"[Title/Abstract] OR "new"[Title/Abstract] OR "novel"[Title/Abstract] OR "initial"[Title/Abstract] OR "experimental"[Title/Abstract] OR "emerging"[Title/Abstract] OR "IDEAL"[Title/Abstract] OR "feasibility"[Title/Abstract] OR "first case"[Title/Abstract] OR "initial case"[Title/Abstract] OR "first in human"[Title/Abstract] OR "1st in human"[Title/Abstract] OR "preliminary"[Title/Abstract] OR "proof of principle"[Title/Abstract] OR "case reports"[Title/Abstract] OR "pilot study"[Title/Abstract]) AND ("invasive"[Title/Abstract] OR "percutaneous"[Title/Abstract] OR "surger*"[Title/Abstract] OR "interventional"[Title/Abstract] OR "surgic*"[Title/Abstract] OR "incision*"[Title/Abstract] OR "operat*"[Title/Abstract]) AND ("humans"[MeSH Terms] OR "human"[Title/Abstract])) NOT (Editorial[pt] OR Review[pt] OR Letter[pt] OR "randomized controlled trial"[Publication Type] OR "RCT"[Title/Abstract] OR "meta-analysis"[Publication Type] OR "commentary"[Publication Type] OR "audit"[Title/Abstract] OR "comparative study"[Publication Type] OR "registry study"[Title/Abstract] OR "economic evaluation"[Title/Abstract] OR "cost-effectiveness"[Title/Abstract] OR "survey"[Title/Abstract] OR "retrospective"[Title/Abstract] OR "animals"[MeSH Terms] OR "drug"[Title/Abstract] OR "medication"[Title/Abstract] OR "pharmaceutical"[Title/Abstract] OR "medicinal"[Title/Abstract]) |
| Web of Science | TS=("early phase" OR "phase I" OR "phase II" OR "innovative" OR "transformative" OR "new" OR "novel" OR "initial" OR "experimental" OR "emerging" OR "IDEAL" OR "feasibility" OR "first case" OR "initial case" OR "first in human" OR "1st in human" OR "preliminary" OR "proof of principle" OR "case reports" OR "pilot study")  AND  TS=("invasive" OR "percutaneous" OR "surger*" OR "surgic*" OR "interventional" OR "incision" OR "operat*")  NOT  TS=("drug" OR "medication" OR "pharmaceutical" OR "medicinal" OR "animal" OR "animals" OR "editorial" OR "review" OR "letter" OR "randomized controlled trial" OR "RCT" OR "meta-analysis" OR "commentary" OR "audit" OR "comparative study" OR "registry" OR "economic evaluation" OR "cost-effectiveness" OR "survey" OR "retrospective") |
| SCOPUS | TITLE-ABS-KEY ( ( "early phase" OR "phase I" OR "phase II" OR "innovative" OR "transformative" OR "new" OR "novel" OR "initial" OR "experimental" OR "emerging" OR "IDEAL" OR "feasibility" OR "first case" OR "initial case" OR "first in human" OR "1st in human" OR "preliminary" OR "proof of principle" OR "case reports" OR "pilot study" ) AND ( "invasive" OR "percutaneous" OR "surger" OR "interventional" OR "surgic" OR "incision" OR "operat" ) AND NOT ( "drug" OR "pharma" OR "medication" OR "medicine" OR "animal study" OR "Editorial" OR "Review" OR "Letter" OR "randomized controlled trial" OR "RCT" OR "meta-analysis" OR "commentary" OR "audit" OR "comparative non-randomised studies" OR "registry study" OR "economic evaluation" OR "survey" OR "retrospective study" ) ) AND PUBYEAR = 2025 AND ( LIMIT-TO ( SUBJAREA , "MEDI" ) OR LIMIT-TO ( SUBJAREA , "AR" ) ) |
