# Supplementary material for: Are equity, diversity and inclusion considered in early-phase studies evaluating innovative and developing surgical procedures? Protocol for a scoping review
Source: BMJ Open. 2026 Feb 9;16(2):e112489. doi: 10.1136/bmjopen-2025-112489 (PMC12887517; doi:10.1136/bmjopen-2025-112489)
Supplement: Supplementary file 2 [file bmjopen-2025-112489.reviewer_comments.pdf]

PEER REVIEW HISTORY

BMJ Open publishes all reviews undertaken for accepted manuscripts. Reviewers are asked to complete a checklist review form and are provided with free text boxes to elaborate on their assessment. These free text comments are reproduced below.

ARTICLE DETAILS

Title (Provisional)

Are equity, diversity and inclusion considered in early phase studies evaluating innovative and developing surgical procedures? Protocol for a scoping review

Authors

Etemadi, Manal; Macefield, Rhiannon; Avery, Kerry; Elliott, Daisy; Dawson, Shoba; Blencowe, Natalie S; Coyle, Maeve; Richards, Hollie Sarah; Graham, Benjamin; Jones, Eddie; Torkington, Amber; Chatfield, Katie; Malik, Hussein; Garlick, Macca; Blazeby, Jane

---

VERSION 1 - REVIEW

**Reviewer**	**1**
**Name**	**Turner , Richard**
**Affiliation**	**University of Tasmania**
**Date**	**01-Nov-2025**
**COI**	**None**

In Table 2, there is an error in one of the exclusion criteria :"Publications in the English language"'

Typo in Table 3" sextual.

Please specify whether trans people are also included under 'sex'; and if so, how will papers describing innovative gender reassignment procedures be dealt with?

**Reviewer**	**2**
**Name**	**Daodu, Oluwatomilayo**
**Affiliation**	**Alberta Health Services Department of Surgery**
**Date**	**11-Nov-2025**
**COI**	**None**

Thank you very much for your submission. This is an important topic that requires attention from the scientific community. The methodology of a scoping review is sensible to answer the research question. However, there are several concerns with this protocol as it is now.

1. There is not at least one search strategy provided from one of the databases mentioned. Therefore it is difficult to evaluate the search strategy that produced over 5000 abstracts and why that may be the case. Additionally, the time frame is extremely limiting (January to Apr 2025), and may be overly restrictive, especially in light of recent political shifts that may have made EDI reporting less desirable.

2. The definitions of EDI are not well articulated and the areas of EDI are not clear at the beginning of the manuscript including what is meant by an "EDI factor". Further, the framing of areas of EDI is problematic in that there is no mention of the structural and systemic contributors that may impact member of equity-deserving groups. For instance, "race/ethnicity" is noted to be a possible exposure. For instance, the phrasing makes race/ethnicity sound like a biological reality rather than a social construct and fails to attend to the structural contributors of differences in outcomes for racialized patients.

3. Similarly, there is a misrepresentation of the references regarding how "wounds may heal differently". The referenced article primarily focuses on patients' perceptions of their wound healing. This is an important distinction, because, again, it is important not to conflate what is biological with what is structural.

4. Single Patient case studies are in the inclusion criteria. How can EDI factors be distinguished in a single patient case study?

5. The screening methodology is not clear. How many reviewers will there be for title/abstract screening? And what is the justification for a single screener for 80%, especially if the individuals screening the remaining 80% are not the same as those for the first 20%. This process should be made more clear.

6. Quality assessment will be performed, but not with a validated tool. Please justify either the use of the non-validated tool or the need for quality assessment in the review.

VERSION 1 - AUTHOR RESPONSE

Reviewer: 1

Comments to the Author:

Comments	Response
In Table 2, there is an error in one of the exclusion criteria: “Publications in the English language"'	Corrected. Non-English.
Typo in Table 3" sextual.	Corrected
Please specify whether trans people are also included under 'sex'; and if so, how	Thank you for this helpful comment. We agree that clarity is needed regarding how we define, and handle data related to sex

will papers describing innovative gender reassignment procedures be dealt with?	and gender diversity. We have now explicitly stated that our review will include studies involving participants. We have also clarified how we will treat studies focusing specifically on gender reassignment procedures.

Reviewer: 2
Comments to the Author:
Thank you very much for your submission. This is an important topic that requires attention from the scientific community. The methodology of a scoping review is sensible to answer the research question. However, there are several concerns with this protocol as it is now.

Comments	Response
1. There is not at least one search strategy provided from one of the databases mentioned. Therefore, it is difficult to evaluate the search strategy that produced over 5000 abstracts and why that may be the case.<br>Additionally, the time frame is extremely limiting (January to Apr 2025), and may be overly restrictive, especially in light of recent political shifts that may have made EDI reporting less desirable.	Thank you for this thoughtful comment.<br>We added the search strategy table which includes separate search strategy for each database.<br>We agree that the short time frame could appear restrictive; however, this decision was made deliberately in light of the very large and continually expanding volume of early phase surgical studies, many of which report “first use” or novel technical adaptations. Restricting the time frame allows us to conduct a detailed and rigorous analysis of EDI reporting within a manageable yet representative contemporary dataset.<br>We also note that the aim of this review is not to provide a trend analysis, but rather to characterise current EDI reporting standards in early phase surgical innovation. Nevertheless, we will record the publication month and year of included studies to allow future updates or comparisons as reporting practices evolve.
2. The definitions of EDI are not well articulated and the areas of EDI are not clear at the beginning of the manuscript including what is meant by an "EDI factor".<br>Further, the framing of areas of EDI is problematic in that there is no mention of the structural and systemic	We agree that greater clarity was needed regarding how we define EDI and what is meant by “EDI factors.” Our scoping review is focused specifically on whether and how EDI dimensions are considered and reported in early phase surgical studies, rather than on explaining or analysing the

contributors that may impact member of equity-deserving groups. For instance, "race/ethnicity" is noted to be a possible exposure. For instance, the phrasing makes race/ethnicity sound like a biological reality rather than a social construct and fails to attend to the structural contributors of differences in outcomes for racialized patients.	structural and systemic determinants that shape inequities.<br>We have, however, clarified that EDI dimensions (e.g., sex, gender, race/ethnicity, disability, socioeconomic position) are socially constructed categories rather than biological variables, and that we will capture how studies conceptualise and report them. This ensures conceptual precision while keeping the review's scope aligned with its stated aim. This text is on page 7.
3. Similarly, there is a misrepresentation of the references regarding how "wounds may heal differently". The referenced article primarily focuses on patients' perceptions of their wound healing. This is an important distinction, because, again, it is important not to conflate what is biological with what is structural.	We have revised the sentence accordingly to more accurately represent the evidence and to align with our focus on EDI considerations in surgical research.<br>The referenced study examines differences in patients' perceptions and experiences of scarring between African American and White participants, including psychosocial and quality-of-life impacts. These findings highlight social and structural contributors, such as access to care, support, and cultural context, rather than biological differences in healing.
4. Single Patient case studies are in the inclusion criteria. How can EDI factors be distinguished in a single patient case study?	Single-patient case studies are valuable for examining how EDI dimensions are acknowledged and represented in surgical innovation research.<br>Specifically, we will extract information on whether and how authors:<br>• Report patient characteristics relevant to EDI (e.g., sex, gender, ethnicity, socioeconomic background, disability status);<br>• Discuss implications for broader populations or for equitable access to the innovation.<br>Thus, for single-patient studies, our analysis will focus on the consideration of EDI factors in reporting and interpretation, rather than on comparative outcomes.
5. The screening methodology is not clear. How many reviewers will there be for title/abstract screening? And what is	We have clarified our screening methodology. Due to the large volume of early-phase surgical studies, dual

the justification for a single screener for 80%, especially if the individuals screening the remaining 80% are not the same as those for the first 20%. This process should be made more clear.	independent screening of all records is not feasible. To ensure consistency and reliability, two reviewers will independently screen an initial 20% of records, aligning inclusion/exclusion criteria. The remaining 80% will be screened by a single reviewer, with any uncertain or borderline studies flagged for discussion with a second reviewer. This approach balances feasibility and methodological rigor and is consistent with established practices in scoping and rapid reviews. All reviewers will be trained to ensure that reliable application of the inclusion criteria is occurring before beginning independent screening.
6. Quality assessment will be performed, but not with a validated tool. Please justify either the use of the non-validated tool or the need for quality assessment in the review.	We originally planned to include a quality assessment; however, upon reflection, we agree that this may not add methodological value. As this is a scoping review, our primary aim is to map how EDI dimensions are considered in early-phase surgical studies, rather than to critically appraise study outcomes or methodological rigor. As the formal quality assessment is not required for scoping reviews, and we have removed this step from the protocol.

VERSION 2 - REVIEW

**Reviewer**	**2**
**Name**	**Daodu, Oluwatomilayo**
**Affiliation**	**Alberta Health Services Department of Surgery**
**Date**	**18-Dec-2025**
**COI**	

Thank you for the thoughtful revisions which add clarity to the review protocol
